# The role of the cytoskeleton in fibrotic diseases

**DOI:** 10.3389/fcell.2024.1490315

**Published:** 2024-10-24

**Authors:** Caoyuan Niu, Yanan Hu, Kai Xu, Xiaoyue Pan, Lan Wang, Guoying Yu

**Affiliations:** ^1^ State Key Laboratory Cell Differentiation and Regulation, Henan International Joint Laboratory of Pulmonary Fibrosis, Henan Center for Outstanding Overseas Scientists of Organ Fibrosis, College of Life Science, Henan Normal University, Xinxiang, China; ^2^ Department of Reproductive Medicine, The Third Affiliated Hospital of Xinxiang Medical University, Xinxiang, China

**Keywords:** fibrosis, cytoskeleton, cell transformation, microfilaments, cytoskeletal remodeling

## Abstract

Fibrosis is the process whereby cells at a damaged site are transformed into fibrotic tissue, comprising fibroblasts and an extracellular matrix rich in collagen and fibronectin, following damage to organs or tissues that exceeds their repair capacity. Depending on the affected organs or tissues, fibrosis can be classified into types such as pulmonary fibrosis, hepatic fibrosis, renal fibrosis, and cardiac fibrosis. The primary pathological features of fibrotic diseases include recurrent damage to normal cells and the abnormal activation of fibroblasts, leading to excessive deposition of extracellular matrix and collagen in the intercellular spaces. However, the etiology of certain specific fibrotic diseases remains unclear. Recent research increasingly suggests that the cytoskeleton plays a significant role in fibrotic diseases, with structural changes in the cytoskeleton potentially influencing the progression of organ fibrosis. This review examines cytoskeletal remodeling and its impact on the transformation or activation of normal tissue cells during fibrosis, potentially offering important insights into the etiology and therapeutic strategies for fibrotic diseases.

## 1 Introduction

Fibrosis is characterized by the excessive proliferation, hardening, and scarring of various tissues due to the over-accumulation of collagen and other extracellular matrix components. This pathological condition results from chronic inflammatory responses to diverse stimuli, including persistent infections, autoimmune reactions, allergic responses, chemical injuries, radiation, and tissue damage. Although current therapies for fibrotic diseases such as idiopathic pulmonary fibrosis, liver cirrhosis, progressive kidney disease, cardiovascular fibrosis, and systemic sclerosis generally focus on targeting inflammatory responses, accumulating evidence indicates that the mechanisms underlying fibrosis are distinct from those governing inflammation (Wynn, 2008). Research indicates that cytoskeletal reorganization plays a crucial role in fibrotic diseases. During the progression of pulmonary fibrosis, the reorganization of the actin cytoskeleton mediated by the ROS/RhoA-ROCK pathway induces myofibroblast transformation and collagen synthesis, ultimately influencing the outcome of lung fibrosis (Ni et al., 2013). Furthermore, studies have demonstrated that the reprogramming of the myofibroblast cytoskeleton through integrin-dependent mechanisms can influence the progression of liver fibrosis. This process is regulated by the Wnt1-induced signaling pathway protein 1 (WISP1) and the myocardin-related transcription factor (MRTF) pathway (Xi et al., 2022). Studies have also indicated that the actin-binding protein developmentally regulated brain protein (Drebrin) can facilitate the fibrosis process in cardiac and hepatic myofibroblasts. It does so by promoting the development of an actin cytoskeleton and the expression of collagen triple helix repeat containing 1 (Cthrc1) (Hironaka et al., 2023). During fibrosis, the activation of the RhoA signaling pathway leads to cytoskeletal remodeling, which in turn activates MRTF. MRTF further promotes the expression of fibrosis-related genes by binding to serum response factor (SRF), activating a series of genes involved in cytoskeletal reorganization and fibrotic mediators. Additionally, MRTF activation strengthens RhoA signaling through a positive feedback loop, with MRTF-dependent upregulation of GEF-H1 mediated in cooperation with the transcription factor Sp1. This reveals the close connection between fibrosis and the cytoskeleton (Venugopal et al., 2024). This review provides a comprehensive overview of the structure and function of the cytoskeleton, with a focus on the alterations observed in the cytoskeleton of pathogenic cells in fibrotic organs. Additionally, it explores the potential of targeting these cytoskeletal changes as a therapeutic strategy for treating fibrotic diseases.

## 2 Cytoskeleton classification and physiological functions

The cytoskeleton is a dynamic fibrous network composed of proteins within the cell. It provides structural support, mechanical strength, and maintains cell polarity. Additionally, the cytoskeleton is involved in various cellular physiological processes, including cell mobility, signal transduction, and the spatial organization of internal organelles (Bezanilla et al., 2015). The cytoskeleton is primarily composed of three structural components: microfilaments, microtubules, and intermediate filaments (IFs). Microfilaments are composed of actin, microtubules are formed from tubulin proteins, and IFs consist of various subunits (Hohmann and Dehghani, 2019; Pollard and Goldman, 2018). Microfilaments, microtubules, and IFs each play distinct roles in various physiological processes and have unique functions. Furthermore, these different types of cytoskeletal elements interact in complex ways to regulate the cell’s overall physiological state (Masters et al., 2017).

Microfilaments primarily maintain cellular morphology and structure, playing a crucial role in cellular architecture, particularly at the cell periphery and cortex (Steffen et al., 2017). Actin forms microfilaments that provide mechanical support and facilitate cell motility. It is involved in various biological processes, such as the extension and contraction of the cell edge, as well as intracellular transport processes within the microtubule system (Pollard and Cooper, 2009).

Microtubules are a fundamental component of the cytoskeleton in eukaryotic cells, playing key roles in cell division, morphology, motility, and intracellular transport. Although their specific functions can vary, microtubules are composed of highly conserved tubulin proteins, which share nearly identical molecular structures. The characteristics and functions of the microtubule cytoskeleton are modulated by various tubulin isotypes and post-translational modifications, a regulatory system referred to as the “tubulin code.” This code is intricately linked to many human physiological and pathological processes (Janke and Magiera, 2020).

IFs consist of one or more members of the cytoskeletal protein family, with their expression being specific to particular cell and tissue types. These filaments are involved in cellular movement and signal transduction, offering mechanical support and maintaining the structural integrity of cells and tissues (Lowery et al., 2015; Herrmann and Aebi, 2016). Vimentin is a prevalent cytoplasmic intermediate filament protein that plays a pivotal role in stabilizing intracellular structures (Paulin et al., 2022).

## 3 Fibrotic diseases

Fibrosis is a physiological and pathological process marked by the abnormal accumulation of collagen and other ECM components within tissues. It plays a crucial role in wound healing and tissue repair, occurring in response to various triggers, including infections, inflammation, autoimmune diseases, degenerative conditions, tumors, and injuries (Wynn and Ramalingam, 2012a). However, excessive formation and deposition of collagen and extracellular matrix, beyond the tissue’s capacity for degradation and metabolism, can result in structural and functional abnormalities in tissues and organs. Such conditions can affect various organs and systems, including the lungs, liver, heart, kidneys, and skin, and may also lead to systemic fibrotic disorders (Fertala et al., 2023).

**FIGURE 1:**
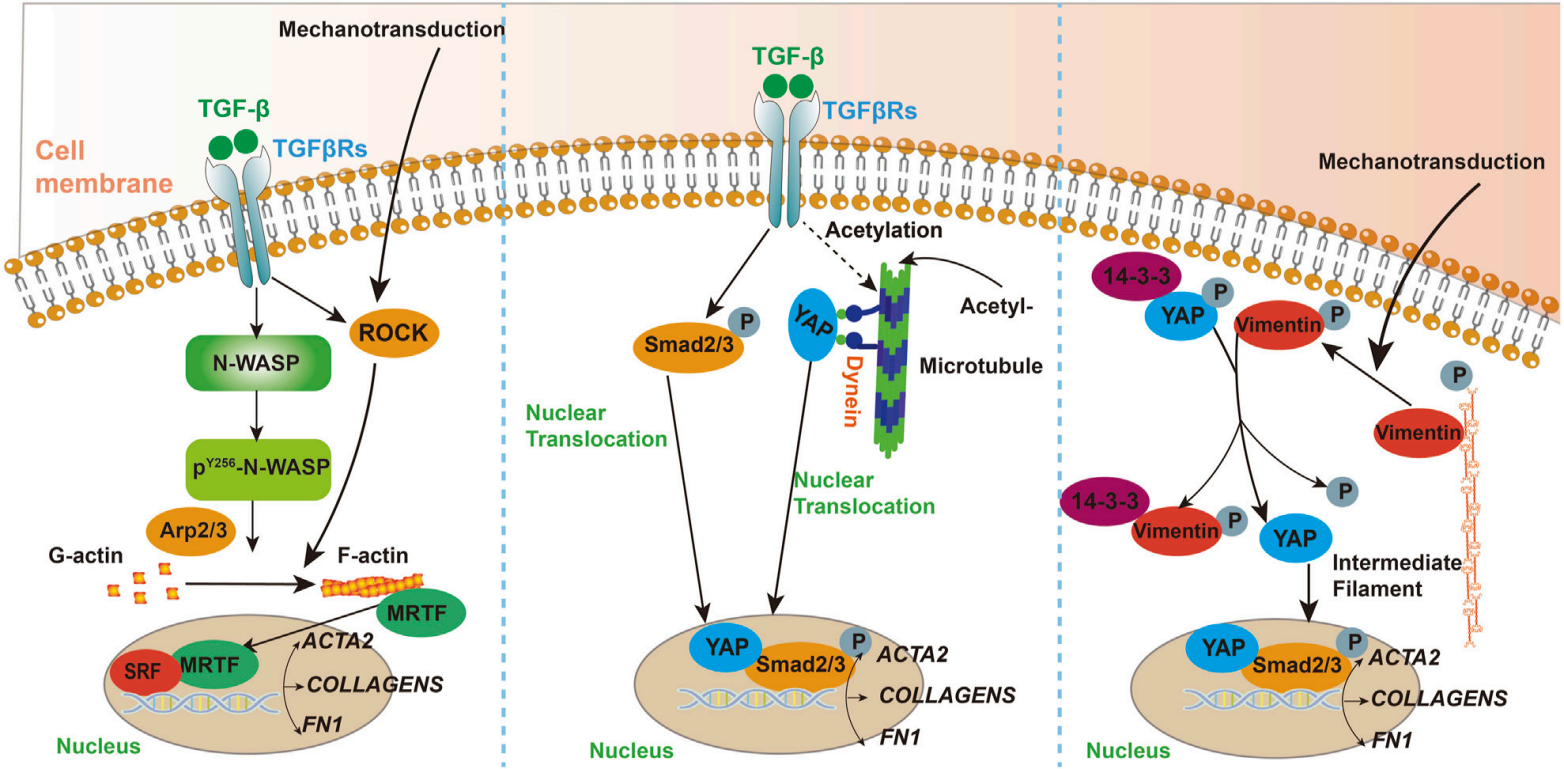
A schematic illustrating the key pathways and mediators involved in the mechanisms of cytoskeletal participation in fibrosis.

The etiology of fibrotic diseases is multifaceted, encompassing a range of factors such as genetic predispositions, environmental exposures, immune system dysfunctions, and chronic inflammation (Spruit et al., 2013). Although different types of fibrotic diseases may have distinct etiologies, it has been established that the cytoskeleton plays a crucial role in the development and progression of these conditions which was presented in Figure 1 (Riches et al., 2015; Niu et al., 2023; You et al., 2020; Li et al., 2017).

### 3.1 Pulmonary fibrosis

Pulmonary fibrosis represents the terminal stage of various acute and chronic lung diseases and is associated with a poor prognosis. The average survival time is typically only 3–5 years after diagnosis (Cheng et al., 2022). The pathological hallmark of pulmonary fibrosis is the abnormal deposition of collagen and extracellular matrix components. While the precise pathogenic mechanisms remain unclear, various factors can induce and exacerbate the progression of fibrosis. These include genetic predisposition, environmental exposures, autoimmune abnormalities, and viral infections (Zhao et al., 2023). These inducing factors cause abnormal cytokine release in lung tissue and adaptive remodeling of tissue structure, ultimately leading to irreversible pulmonary fibrosis through the combined effects of multiple factors (Liu et al., 2021).

Transforming Growth Factor Beta 1 (TGF-β1) plays a critical role in the development and progression of pulmonary fibrosis. Moreover, TGF-β1 is vital for the formation of smooth muscle actin filaments during myofibroblast differentiation, a process facilitated by neural Wiskott-Aldrich syndrome protein (N-WASP) (Cai et al., 2012). Research has shown that TGF-β1 can promote the assembly of cellular actin filaments and increase cellular tension, thereby accelerating the progression of experimental pulmonary fibrosis (Niu et al., 2023). The microtubule-destabilizing agent Fenbendazole has been shown to alleviate experimental fibrosis by disrupting microtubule-dependent energy metabolism in fibroblasts and attenuating TGF-β-induced fibroblast activation (Wang et al., 2022). Rho-associated coiled-coil kinase (ROCK) has been implicated in the differentiation of myofibroblasts in response to changes in matrix stiffness under fibrotic conditions. Studies have demonstrated that knockout of either ROCK1 or ROCK2 leads to a significant reduction in α-SMA expression on soft experimental matrices, suggesting that ROCK contributes to the progression of fibrosis by modulating cellular tension in fibroblasts (Htwe et al., 2017). In hyperoxia-induced pulmonary fibrosis, actin cytoskeleton rearrangement plays a key role. Hyperoxia promotes the differentiation of pulmonary fibroblasts into myofibroblasts, marked by increased α-smooth muscle actin (α-SMA) synthesis. The RhoA-ROCK signaling pathway enhances this process, affecting myofibroblast differentiation and collagen synthesis. Animal studies suggest that RhoA and ROCK inhibitors, as well as ROS scavengers, could be potential strategies for preventing and treating oxygen toxicity-induced pulmonary fibrosis (Ni et al., 2013).

In the formation of alveolar septa, the remodeling of the cytoskeleton in lung fibroblasts (LFs) is crucial for increasing the gas exchange surface area (McGowan et al., 2024). The Drebrin protein plays a key role in regulating the cytoskeleton of LFs, particularly in balancing cell contraction and migration. Research indicates that the absence of Drebrin leads to a reduction in alveolar surface area and an enlargement of alveolar ducts, highlighting its importance for normal alveolar development. Additionally, LFs lacking Drebrin exhibit defects in response to matrix stiffness and migration speed, along with impaired activity of non-muscle myosin-2 (NM2) (McGowan et al., 2024). These findings provide new insights into the mechanisms underlying emphysema and pulmonary fibrosis, suggesting that abnormal regulation of the cytoskeleton may be a key factor in these diseases.

### 3.2 Hepatic fibrosis

Hepatic fibrosis is a chronic liver condition marked by excessive fibrous tissue formation, resulting in structural and functional abnormalities in the liver (Wallace et al., 2008). Hepatic fibrosis is an early stage of cirrhosis that is potentially reversible. Without timely intervention, it can progress to irreversible cirrhosis. The development of hepatic fibrosis is linked to chronic viral hepatitis, alcohol abuse, fatty liver disease, and exposure to drugs and toxins (Yang et al., 2022).

Activated hepatic stellate cells are crucial in the pathogenesis of hepatic fibrosis, serving as the primary source of myofibroblasts (Krenkel et al., 2018). Research indicates that activated hepatic stellate cells (HSCs) play a pivotal role in the progression of hepatic fibrosis. The rearrangement of the F-actin cytoskeleton is closely linked to the activation of HSCs. Inhibiting the rearrangement of actin filaments in these cells may suppress the development of liver fibrosis and cirrhosis (Cui et al., 2014). Activated hepatic stellate cells are also capable of promoting cytoskeletal remodeling and facilitating cell migration (Elnagdy et al., 2023a). Studies have demonstrated that a deficiency in α-smooth muscle actin (α-SMA) results in a reduction of hepatic fibrosis. This decrease in fibrosis is attributed to the disruption of cytoskeletal signaling within hepatic stellate cells (Rockey et al., 2019). During the process of liver fibrosis, hepatic stellate cells (HSCs) and fibroblasts are activated, producing large amounts of extracellular matrix (ECM), especially collagen, leading to liver structural and functional damage. Microtubule acetylation enhances the activity of the TGF-β1/Smad signaling pathway by promoting the interaction between YAP protein and microtubules as well as its nuclear translocation, further driving fibroblast activation and differentiation. Dynein, a microtubule motor protein, is involved in this process, and increased activity of α-Tubulin acetyltransferase 1 (α-TAT1) is associated with elevated microtubule acetylation levels, collectively contributing to the progression of liver fibrosis. Investigating these roles of microtubules can help elucidate the molecular mechanisms of liver fibrosis and provide potential targets for developing new therapeutic strategies (You et al., 2020).

In summary, during the development of hepatic fibrosis, the rearrangement of actin filaments in hepatic stellate cells, triggered by various factors, promotes cell activation. This results in the accumulation of myofibroblasts and excessive production of collagen and extracellular matrix, leading to liver fibrosis. As fibrosis progresses, it can lead to cirrhosis. Therefore, targeting actin filament remodeling could be a potential therapeutic approach for treating liver fibrosis.

### 3.3 Renal fibrosis

Renal fibrosis, a process linked to chronic kidney disease, is characterized by the excessive formation of fibrous tissue within the kidney. This results in structural and functional abnormalities in nephron units (Li et al., 2022). Renal fibrosis is a common outcome in various chronic kidney diseases, including chronic glomerulonephritis, hypertensive nephropathy, and diabetic nephropathy, potentially leading to kidney function loss. The primary causes include chronic glomerulonephritis, hypertensive nephropathy, and diabetic nephropathy. The mechanisms underlying renal fibrosis involve chronic inflammation, abnormal cytokine expression, and extracellular matrix deposition (Zhou et al., 2023; Huang et al., 2018; Du et al., 2023).

It has been reported that in experimental renal fibrosis induced by asymmetric dimethylarginine (ADMA), ADMA promotes the accumulation of stress fibers, enhances NF-κB DNA binding, and increases TGF-β1 expression. Disruption of actin dynamics using the actin-depolymerizing agent cytochalasin D, the actin-stabilizing agent jasplakinolide, or removal of stress fiber bundles through the NADPH oxidase inhibitor apocynin and the p38 MAPK inhibitor SB203580 significantly suppresses ADMA-induced NF-κB and TGF-β1 DNA binding. These findings suggest that targeting the actin cytoskeleton could be a promising therapeutic strategy for renal fibrosis (Wang et al., 2019). In diabetic kidney injury-induced renal fibrosis, the actin cytoskeleton-mediated nuclear translocation of NF-κB is critical for activating the pro-fibrotic factor Rho kinase. Gene knockout studies have highlighted the essential role of ROCK2 in the progression of diabetic kidney injury. Consequently, targeting cytoskeletal alterations driven by glomerular ROCK2 may offer a promising therapeutic strategy for diabetic nephropathy and associated fibrosis (Yosuke Nagai et al., 2019). In polycystic kidney disease, the loss of function in Polycystin1 or Polycystin2 activates the RhoA signaling pathway, leading to cytoskeletal reorganization and the nuclear translocation of MRTF. This promotes the production and secretion of fibrotic mediators, including connective tissue growth factor (CTGF). These changes not only affect the morphology and function of epithelial cells but also activate surrounding mesenchymal cells and the fibrosis process through paracrine signaling, collectively driving the development of renal fibrosis (Lichner et al., 2024).

In summary, during renal fibrosis, reprogramming of the actin cytoskeleton enhances NF-κB nuclear translocation and Rho kinase activation, promoting NF-κB and TGF-β1 DNA binding and advancing fibrosis progression. Thus, targeting the actin cytoskeleton offers a significant therapeutic strategy for kidney injury and fibrosis. Developing drugs that address these pathways could help mitigate kidney damage and slow fibrosis progression.

### 3.4 Cardiac fibrosis

Cardiac fibrosis is a pathological process marked by alterations in cardiomyocytes, cardiac fibroblasts, and the collagen I/III ratio. It involves excessive ECM production and deposition, leading to scar tissue formation, structural changes in the heart, and impaired systolic and diastolic function. Cardiac fibrosis is prevalent in advanced cardiovascular diseases such as ischemic heart disease, hypertension, and heart failure (Qin et al., 2021). Cardiac fibrosis often results from an inflammatory response or damage repair. Following injury, cardiomyocyte loss triggers adverse myocardial remodeling and fibrosis, impairing the heart’s contraction and pumping function and leading to heart failure (Aharonov et al., 2020).

Actin assembly plays a role in promoting the development of cardiac fibrosis. Additionally, a novel molecule, yes-associated protein (YAP) circular RNA (circYap), has been identified. CircYap influences cardiac remodeling during fibrosis by modulating actin polymerization, indicating its potential as a therapeutic target for future treatment of cardiac fibrosis (Wu et al., 2021). Myocardial fibrosis is driven by inflammation. Single-cell analysis identifies Drebrin, an actin-binding protein, in fibroblasts of fibrotic heart tissue. Drebrin enhances actin cytoskeleton formation and signaling of MRTFs and SRF, exacerbating fibrosis. Targeting Drebrin may offer a novel therapeutic strategy (Hironaka et al., 2023). A study on cardiac valve fibrosis revealed that high FBN1 and low MMP2 levels are linked to severe mitral fibrosis. Additionally, decreased FLNA expression with age may protect against valve aging, while SOX9 appears to reduce calcification risk. Inflammation may further promote fibrosis by affecting cytoskeletal and extracellular matrix interactions, underscoring the significant role of cytoskeletal proteins in valve fibrosis (Opris et al., 2024). SGLT2 inhibitors reduce cardiovascular fibrosis by inhibiting endothelial-to-mesenchymal transition (EndMT) and regulating cardiac fibroblast activation. They alter cytoskeletal protein expression, affecting fibroblast proliferation and migration. Additionally, SGLT2 inhibitors target transporters like NHE1, SMIT, and SMVT, which maintain cytoskeletal integrity, further inhibiting fibrosis progression (Schmidt et al., 2024).

During cardiac fibrosis, remodeling of the actin cytoskeleton accelerates the disease’s progression. Consequently, targeting the actin cytoskeleton presents a critical intervention point for cardiac damage and fibrosis. Therapeutic agents that modulate cytoskeletal remodeling may help mitigate cardiac damage and slow the advancement of cardiac fibrosis.

### 3.5 Ocular fibrosis

Ocular fibrosis is characterized by the excessive proliferation and deposition of fibrous connective tissue within the eye, resulting in structural and functional abnormalities in ocular tissues (Saika et al., 2008). This process can affect multiple ocular tissues and structures, including the cornea, sclera, conjunctiva, and peri-lenticular tissues. Fibrosis in these areas may impair vision and overall ocular health (Friedlander, 2007). Ocular fibrosis can arise from various factors, including eye injuries, inflammation, infections, and autoimmune diseases (Hinz, 2016; Stepp and Menko, 2021; Ludgate, 2019). Both addressing the underlying cause and managing symptoms can benefit patients with ocular fibrosis.

Research indicates that selectively inhibiting actin cytoskeleton remodeling in endothelial cells reduces experimental choroidal neovascularization and subretinal fibrosis. This suggests that actin remodeling in choroidal endothelial cells promotes neovascularization and subretinal fibrosis (Caballero et al., 2011). Damage to lens epithelial cells (LECs) can induce epithelial-mesenchymal transition (EMT) and lead to lens fibrosis. Tropomyosin proteins, which regulate and stabilize actin, are crucial in this process. Abnormal tropomyosin expression disrupts LEC integrity and cellular physiology. Targeting actin cytoskeleton remodeling in LECs thus represents a promising strategy for treating lens fibrosis (Kubo et al., 2012). In corneal fibroblasts, vimentin is crucial for maintaining cell morphology and regulating proliferation and migration in response to PDGF. It plays a key role in cytoskeletal remodeling during myofibroblast differentiation and is essential for generating traction forces for matrix remodeling, a process linked to fibrosis. Proteomic analysis in vimentin-deficient cells suggests its involvement in compensatory mechanisms during corneal fibroblast fibrosis (Miron-Mendoza et al., 2024).

Structural damage to optical pathways—such as the cornea, lens, vitreous body, and retina—can trigger cellular repair processes. Uncontrolled repair may lead to fibrosis. Actin fiber remodeling can drive neovascularization or EMT, contributing to tissue fibrosis. Therefore, drugs targeting actin remodeling offer a novel approach for treating ocular fibrosis.

### 3.6 Intestine fibrosis

Intestinal fibrosis is a common complication of chronic intestinal diseases, resulting from the proliferation of fibroblasts and excessive extracellular matrix (ECM) deposition after chronic inflammation. This condition leads to narrowing of the intestinal lumen and functional disruption. Current treatments, including anti-inflammatory drugs, have limited success, with surgery often being the primary option. The progression of fibrosis involves the interaction of inflammatory cells, cytokines, ECM, epithelial-mesenchymal transition (EMT), fibroblast differentiation, and the gut microbiome. Ongoing research is exploring new therapeutic strategies targeting these mechanisms, such as ECM modulation, cytokine inhibition, and TGF-β pathway targeting (Liu et al., 2024).

Research shows ROCK is activated in inflamed and fibrotic intestinal tissues, playing a critical role in fibrosis related to inflammatory bowel disease (IBD). The ROCK inhibitor AMA0825 reduces fibrogenic factor production by inhibiting TGF-β1-induced activation of MRTF and p38 MAPK while promoting autophagy in fibroblasts. It effectively reverses established intestinal fibrosis and lowers pro-fibrotic protein secretion from Crohn’s disease stenotic tissues (Holvoet et al., 2017).Keratin intermediate filaments play a significant role in intestinal fibrosis through multiple mechanisms, including providing structural support to cells and maintaining barrier function, participating in cell signaling and regulating cell differentiation and proliferation, serving as mediators and biomarkers of inflammatory responses, and engaging in interactions with the microbiome and cellular energy metabolism. These combined effects make keratin a key factor influencing the progression of intestinal fibrosis and provide potential targets for the development of therapeutic strategies (Polari et al., 2020).

Targeting candidate sites such as ROCK or other pathways involved in cytoskeletal organization, epithelial-mesenchymal transition (EMT), and autophagy for the treatment of intestinal fibrosis may disrupt conventional approaches to managing intestinal fibrosis and open new avenues for therapy.

### 3.7 Other organ fibrosis

In addition to pulmonary, hepatic, renal, cardiac, and ocular fibrosis, fibrosis can impact other organs and tissues, including peritoneal fibrosis (thickening and scarring of the abdominal lining), skin fibrosis, meningeal fibrosis, skeletal muscle fibrosis, smooth muscle fibrosis, and pancreatic fibrosis (Raby et al., 2017; Odell et al., 2022; Enos et al., 2019; Zhuang et al., 2023; Ibarrola et al., 2023; Swain et al., 2022). These fibrosis types in various tissues and organs involve excessive fibrous tissue proliferation and deposition, leading to structural damage and functional abnormalities (Henderson et al., 2020).

Different fibrosis diseases have unique causes and mechanisms, but the cytoskeleton is crucial in their development. In peritoneal fibrosis models, Lysophosphatidic Acid Receptor (LPA1) receptor activation in mesothelial cells induces CTGF expression, promoting fibroblast proliferation via a paracrine mechanism. This process drives peritoneal fibrosis through cytoskeletal remodeling (Sakai et al., 2013). TGF-β1 mediates fibroblast activation in fibrosis, including systemic sclerosis (SSc). Engrailed-1 (EN1) is re-expressed in SSc fibroblasts and amplifies TGF-β1 signaling during myofibroblast differentiation. EN1 is induced by TGF-β1 via Smad3 and drives pro-fibrotic effects through ROCK activation and cytoskeletal remodeling. EN1’s role in myofibroblast differentiation is confirmed by functional assays, and fibroblast-specific En1 knockout mice show reduced myofibroblast transition and improved experimental fibrosis outcomes (Györfi et al., 2021). In vascular smooth muscle fibrosis, TRPC6 is essential in the TGF-β1 signaling pathway. Antisense RNA targeting TRPC6 mitigates TGF-β1-induced fibrosis by decreasing myosin light chain phosphorylation, actin stress fiber formation, and cell migration (Park et al., 2017).

Cytoskeletal remodeling impacts fibrosis across various organs, including the lungs, liver, kidneys, heart, eyes, peritoneum, skin, and vascular smooth muscle. Targeting the cytoskeleton presents a potential strategy for developing novel anti-fibrotic drugs to benefit patients.

## 4 Cells involved in fibrosis

**FIGURE 2:**
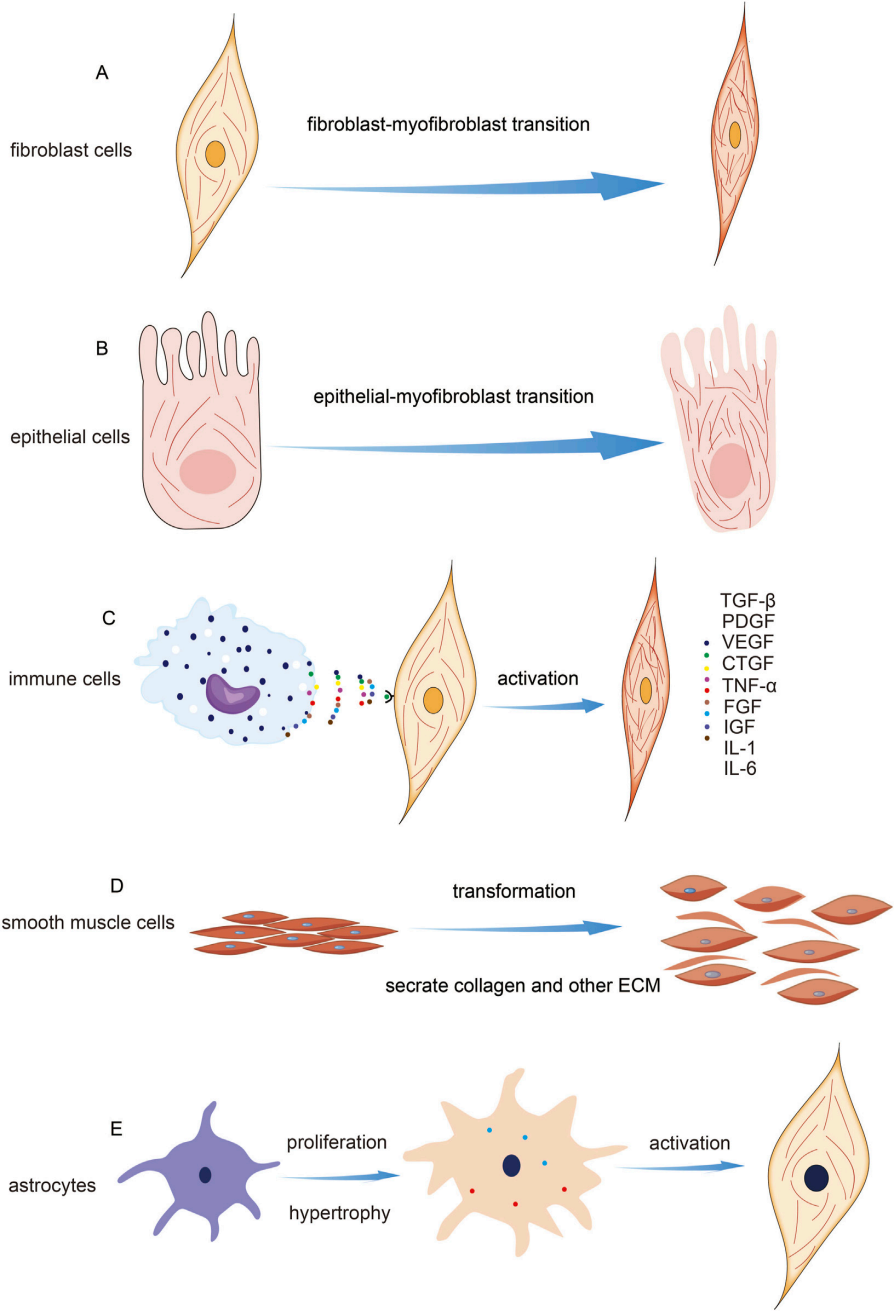
Cell Types Involved in Fibrosis and Their Mechanisms. **(A)** Indication of fibroblast activation and microfilament change. **(B)** Indication of epithelial mesenchymal transition. **(C)** Schematic diagram of fibroblast activation induced by immune cells. **(D)** The transformation of smooth muscle cells during fibrosis. **(E)** The transformation of stellate cells occurs during fibrosis.

Fibrosis is a multifaceted physiological and pathological process that involves various cell types, including fibroblasts, epithelial cells, inflammatory cells, macrophages, smooth muscle cells, and stellate cells (Henderson et al., 2020; Wynn and Ramalingam, 2012b). The cell types involved in fibrosis and their associated pathways are depicted in Figure 2.

### 4.1 Fibroblasts

Fibroblasts are a type of mesenchymal cell widely found in connective tissues such as the skin, lungs, skeletal muscles, and heart. They are a primary cell component of connective tissues and play a variety of important pathological and physiological roles. In the process of fibrosis, fibroblasts are one of the key cell types involved (Plikus et al., 2021). Fibroblast activation is a crucial step in the fibrosis process across different types of fibrotic diseases. Activated fibroblasts are responsible for synthesizing and depositing collagen, fibronectin, and other collagenous proteins, which contribute to the formation of fibrous connective tissue (Qian et al., 2023; Arvia et al., 2022; Geng et al., 2022; Han et al., 2023; Sun et al., 2021; Zhang et al., 2021). In a mouse model of peritoneal fibrosis, researchers have found that LPA and LPA1 play a central role in the proliferation of fibroblasts. The research revealed the critical role of LPA1-induced cytoskeleton reorganization in promoting the fibrotic process and offered new targets for the treatment of fibrotic diseases (Sakai et al., 2013). Another study on IPF explored the role of vimentin intermediate filaments (VimIFs) in regulating fibroblast invasiveness. Findings indicated that increased expression and organization of VimIFs in IPF tissues are linked to autophagy defects. Vimentin is a crucial cytoskeletal protein associated with heightened invasiveness, and its dynamic changes are essential for cell migration. In fibroblasts at the IPF lung tissue margins, vimentin levels were significantly upregulated. The plant-derived compound Withaferin A (WFA) was shown to inhibit VimIF assembly, enhancing autophagy and reducing fibroblast invasiveness. Furthermore, WFA diminished fibroblast invasiveness in 3D lung organoids from IPF patients and protected mice from bleomycin-induced pulmonary fibrosis by promoting autophagy. These results highlight the dual role of VimIFs in IPF and suggest that targeting VimIF assembly could provide an effective strategy for reducing fibroblast invasiveness in pulmonary fibrosis (Surolia et al., 2019).

### 4.2 Epithelial cells

Epithelial cells are a type of cell that lines the surfaces of the body’s external and internal cavities. They constitute the primary component of epithelial tissue. Epithelial tissue performs various important functions in the body, including providing barrier protection, facilitating substance absorption, and enabling structural remodeling (Proud and Leigh, 2011). Epithelial cells participate in the fibrosis process through various mechanisms, including their transformation into mesenchymal cells, promotion of fibroblast activation, involvement in matrix remodeling, and contribution to inflammatory responses. The role of epithelial cells in fibrosis may depend on the specific tissue and pathological context, but their involvement has a significant impact on the occurrence and progression of fibrosis (Banales et al., 2019; Ji et al., 2016; Kalluri and Weinberg, 2009; Kang et al., 2014; Selman, 2006). The remodeling of the cytoskeleton is crucial for the morphological changes observed in alveolar epithelial cells and fibroblasts during fibrosis. TUFT1 enhances microfilament assembly and stress fiber formation through its interaction with N-WASP, which increases cellular contractility and migration. N-WASP promotes the polymerization of branched actin filaments, and TUFT1 facilitates the formation of its activated form, p^Y256^N-WASP, which is vital for TGF-β1-mediated fibroblast activation. Additionally, elevated TUFT1 expression correlates with increased levels of fibrosis markers such as α-SMA, collagen I, and fibronectin, highlighting its significant role in the fibrotic process alongside cytoskeletal remodeling (Niu et al., 2023).

### 4.3 Immune cells

Immune cells play a crucial role in the fibrosis process, including both innate and adaptive immune cells. They release inflammatory mediators and cytokines that promote fibroblast activation and the fibrotic response (Cui et al., 2023; Dhana et al., 2018; Gieseck et al., 2017; Hammerich and Tacke, 2023; Koda et al., 2021; Rurik et al., 2021; Sobecki et al., 2022). Similarly, macrophages are a key cell type in the immune system, involved in clearing damaged tissues and pathogens, and regulating inflammation and repair processes. During fibrosis, macrophages can secrete pro-fibrotic factors that stimulate fibroblast proliferation and collagen synthesis (Lee et al., 2017; Qing et al., 2022; Wynn et al., 2013; Zhang et al., 2022). And macrophages can sense the mechanical properties of the ECM through cytoskeletal remodeling, independent of integrin signaling. This ability allows them to regulate specific gene expression programs related to tissue repair, thereby influencing the fibrosis process (Meizlish et al., 2024). The selective ROCK2 inhibitor GV101 alleviates liver fibrosis by modulating cytoskeletal dynamics and key signaling pathways. It reduces fibrosis by inhibiting pCofilin, impacting actin cytoskeleton remodeling, and targeting the Akt-mTOR-S6K signaling axis, which regulates fibroblast activation, inflammation, and metabolism. These mechanisms collectively contribute to a reduction in collagen levels and fibrotic markers, underscoring GV101’s potential as a therapeutic agent for liver fibrosis (Zanin-Zhorov et al., 2023).

### 4.4 Smooth muscle cells

Smooth muscle cells are a crucial type of muscle cell, primarily distributed in various organs and tissues throughout the body, such as blood vessels, the digestive tract, the respiratory tract, the urinary tract, and the reproductive system. These cells play a role in regulating blood vessel contraction and dilation, as well as tissue repair and remodeling (Donadon and Santoro, 2021). In certain situations, smooth muscle cells may become activated and participate in the fibrosis process, leading to smooth muscle hyperplasia and tissue remodeling (Elamaa et al., 2022; Sun et al., 2023; Zou et al., 2022). The AP-1 family member Jun B regulates visceral smooth muscle cell (SMC) contraction by modulating actin polymerization and myosin light chain phosphorylation. Jun B levels are specifically linked to alterations in the F-actin ratio, which subsequently affects the organization of microfilaments. These results suggest that Jun B plays a crucial role in the regulation of SMC contractility and may contribute to fibrosis development. Targeting Jun B could offer novel therapeutic strategies for treating fibrosis (Ramachandran et al., 2013).

### 4.5 Stellate cells

HSCs are multifunctional cells commonly found in neural tissue and the liver. During liver fibrosis, star-shaped cells can become activated and transform into fibroblasts. They then participate in collagen synthesis and deposition, contributing to the fibrotic process (Anderson et al., 2016; Mohamed Suhaimi and Zhuo, 2012). Studies have shown that HSCs can promote liver fibrosis through cytoskeletal remodeling, facilitating cell migration and activation into myofibroblasts. Targeting the cytoskeletal remodeling of HSCs offers a potential avenue for developing new treatments for liver fibrosis (Elnagdy et al., 2023b).

Overall, the involvement of multiple cell types is a key feature of the fibrotic process. These cells, through their interactions and regulation of signaling pathways, collectively promote or inhibit fibrotic responses and play a crucial role in the remodeling of tissue structure and function.

## 5 Regulation of cytoskeletal dynamics

The cytoskeleton in non-muscle cells comprises a network of fibrous structures regulated by various factors, affecting their assembly and disassembly (Goode et al., 2023; Goodson and Jonasson, 2018; Zhu et al., 2019). To understand its role in fibrotic diseases, we review physicochemical and biological factors impacting microfilaments, microtubules, and IFs. The following Table 1 presents the factors influencing different types of cytoskeleton.

**TABLE 1:** Factors influencing different types of cytoskeleton.

Factors	Microfilaments	Microtubules	Intermediate filaments
physics	Mechanical Stress	Mechanical Stress, Temperature	Mechanical Stress
chemistry	Extracellular matrix compound, G-actin, ATP, Ca^2+^, Mg^2+^, K^+^ Cytochalasin D,Phalloidin Jasplakinolide	Extracellular matrix compound, Colchicine, Taxol, Nocodazole, α-tubulin &β-tubulin, GTP, Mg^2+^	Extracellular matrix compound, pH
biology	small GTPase, Phosphorylation, Arp2/3 Formin, Capping Protein, ADF/Cofilin, α-Actinin and Fimbrin, Thymosin β4 and Profilin	small GTPase, Phosphorylation, γ-TuRC, MAPs, Katanin, Stathmin	small GTPase, Phosphorylation, Gene, sHSPs, interaction of IF and IF, interaction of IF and Actin/microtubule

### 5.1 Microfilaments

Microfilaments, or actin filaments, maintain cell shape, facilitate movement, division, and endocytosis. Their assembly and disassembly are influenced by physical, chemical, and biological factors, which modulate the equilibrium between G-actin and F-actin, impacting cellular functions. Certain drugs affect actin filament dynamics. Cytochalasins bind and sever microfilaments, preventing new monomer addition without affecting disassembly, leading to reduced cell stiffness and volume (Shu et al., 2024). Cylindrocarpine stabilizes microfilaments by preventing disassembly, inhibiting cell movement and used for staining cell morphology (Wagner et al., 2023). Actin-binding proteins regulate actin filament organization and function. Over 100 distinct actin-binding proteins have been isolated, categorized based on their functions. Actin monomer-binding proteins regulate filament assembly. Thymosin β4 inhibits polymerization by blocking polymerization sites, crucial for cell motility and morphology (Pollard, 2016; Cassimeris et al., 1992; Skruber et al., 2018). Profilin regulates actin dynamics by enhancing ATP dissociation from G-actin, supporting rapid cytoskeletal reorganization essential for cell movement and proliferation (Carlier et al., 1993). Nucleating proteins initiate actin filament formation by catalyzing monomer polymerization, organizing the actin cytoskeleton (Dahlstroem et al., 2023). Arp2/3 complex, Formin family, and N-WASP are common nucleating proteins. Cap proteins, like CapZ and α-actinin, regulate filament assembly and stability by binding to filament ends, adapting to cellular changes (Berger et al., 2022). Cross-linking proteins stabilize and connect actin filaments, influencing cellular structure and function. α-Actinin and fimbrin/plastin are notable examples (Wu et al., 2001). Severing and depolymerizing proteins regulate filament remodeling, influencing cell shape and motility. ADF and cofilin are examples, facilitating filament disassembly and restructuring (Inada, 2017; Grintsevich et al., 2016; Larson et al., 2005).

The Rho-ROCK signaling pathway activates RhoA, promoting myosin light chain phosphorylation and increasing actin-myosin interactions, which contribute to fibrotic changes. Additionally, myocardin-related transcription factors translocate to the nucleus upon dissociating from G-actin, activating the transcription of fibrotic genes such as fibronectin and procollagen-1. These dynamics regulate fibroblast activation and proliferation, resulting in excessive deposition of extracellular matrix components, thereby driving the progression of fibrosis (Riches et al., 2015).

### 5.2 Microtubules

Microtubules, composed of α-tubulin and β-tubulin dimers, maintain cell shape, facilitate intracellular transport, support cell division, and mediate signal transduction. Factors affecting microtubule dynamics include temperature, binding proteins, chemical agents, and mechanical stress. Temperature impacts microtubule dynamics; lower temperatures reduce polymerization and depolymerization rates, leading to shorter microtubules (Li and Moore, 2020). Drugs like colchicine, vinblastine, and paclitaxel affect microtubule growth and stability by targeting tubulin proteins, providing insights into microtubule regulation (Wilson et al., 1999). Microtubule-binding proteins (MAPs) aid in microtubule assembly, stability, and interactions within the cytoskeleton, regulated by post-translational modifications and cell-type specificity (Maccioni and Cambiazo, 1995).

Research has shown that microtubules respond to changes in matrix stiffness through the acetylation of α-tubulin at K40, enhancing microtubule stability and promoting the translocation of YAP protein from the cytoplasm to the nucleus. This process activates the YAP signaling pathway, regulating fibroblast activation, migration, and extracellular matrix deposition. These findings reveal the critical role of microtubules in the formation of skin fibrosis and provide a theoretical basis for developing therapeutic strategies targeting α-tubulin acetylation (Wen et al., 2020).

### 5.3 Intermediate filaments

IFs maintain cell shape, mechanical stability, and tissue integrity, influenced by gene expression, protein interactions, cellular signaling, and environmental conditions (Dutour-Provenzano and Etienne-Manneville, 2021). Intermediate filament protein (IFPs) gene expression regulates protein assembly, influencing their diversity and characteristics. Specific genes dictate filament organization, providing mechanical support and influencing cell signaling (Coulombe et al., 2001). Protein interactions, primarily involving unstructured regions, regulate assembly and function. Head domains facilitate tetramer formation, while tail regions influence filament bundling and network organization (Kornreich et al., 2015). IFs regulate critical physiological processes by interacting with signaling molecules, functioning as phosphorylation buffers, and modulating cell-specific signaling activities (Pallari and Eriksson, 2006). IFPs are crucial for stress response, mechanical stability, and biological processes, with dysregulation linked to diseases. They play protective roles in various tissues and contribute to cellular osmotic balance and mechanical signal transduction (Pekny and Lane, 2007; Leube et al., 2015). IFPs modulate cell mechanical properties and signaling during the cell cycle, particularly during cell division. Phosphorylation by kinases facilitates mitotic progression by altering IF organization (Dutour-Provenzano and Etienne-Manneville, 2021).

The functions of intermediate filaments in the cytoskeleton are often underestimated, however, they play a significant role in the process of fibrosis. Vimentin intermediate filaments play a critical role in the process of fibrosis. They are involved not only in regulating the proliferation of mesenchymal cells and collagen deposition but also in reducing the severity of pulmonary fibrosis in Vim^−/−^ mouse models where vimentin is absent. Furthermore, the increased expression of vimentin is associated with inflammatory responses, which represent an important early stage in the development of fibrosis. Vimentin also promotes collagen synthesis and deposition by maintaining the stability of collagen mRNA, thereby affecting organ dysfunction. Therefore, vimentin is a potential therapeutic target for fibrosis, and the development of drugs targeting vimentin may help modulate tissue repair responses, thereby preventing or alleviating the progression of fibrosis (Surolia and Antony, 2022).Research has shown that vimentin plays a crucial role in fibrosis, with its expression increasing in fibrotic tissues. This increase is associated with mesenchymal cell expansion and collagen deposition, promoting the process of epithelial-mesenchymal transition (EMT). Studies using Vim^−/−^ mouse models indicate that vimentin is essential for the development of pulmonary fibrosis, as its absence can alleviate the degree of fibrosis in mice. Furthermore, vimentin is involved in regulating cell migration and matrix remodeling, both of which are critical processes in fibrosis. Therefore, vimentin not only drives the progression of fibrosis but may also serve as a potential therapeutic target for fibrosis-related diseases (Ridge et al., 2022).

**FIGURE 3:**
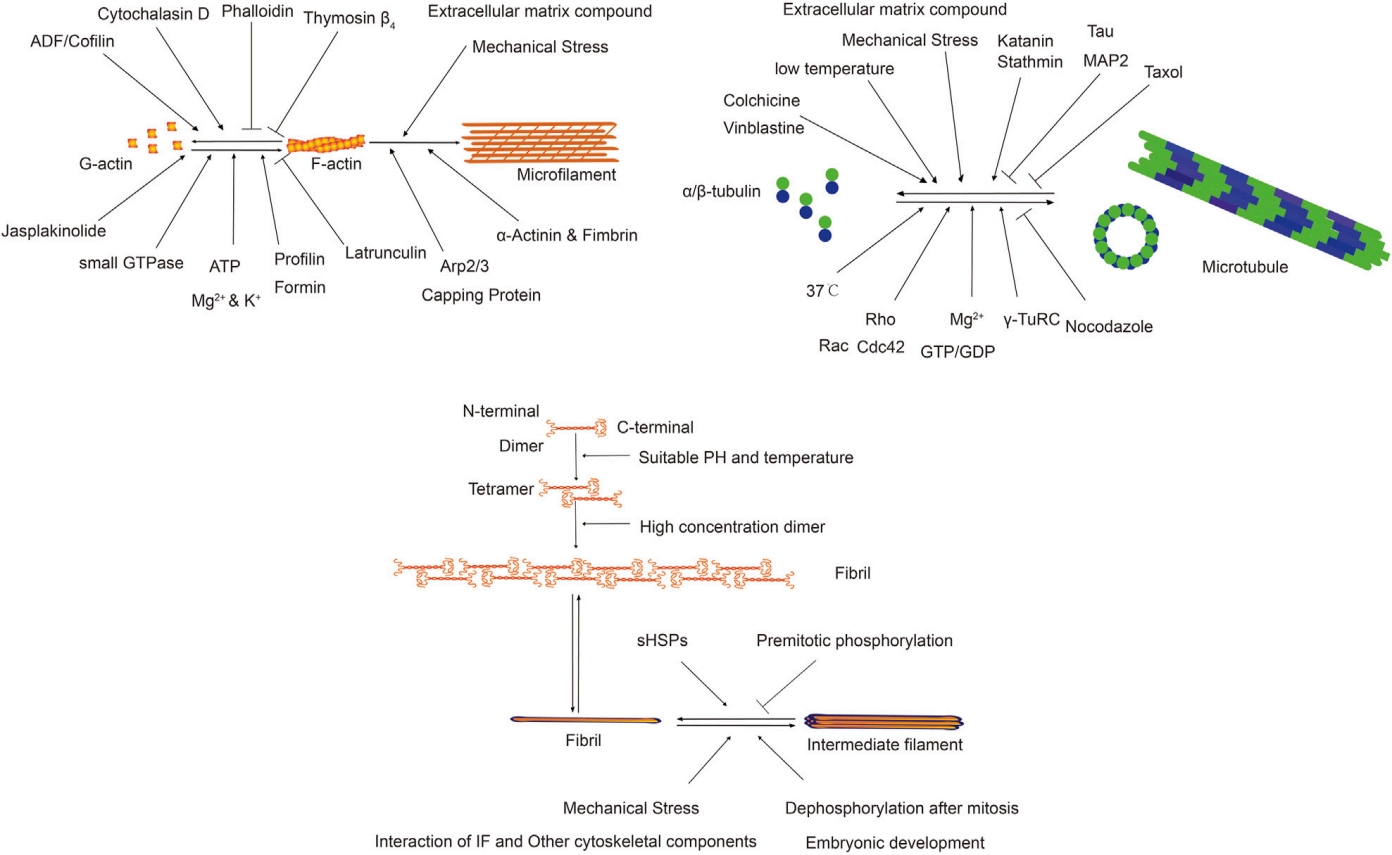
Factors influencing different types of cytoskeleton and their functions.

The following Figure 3 illustrates the factors influencing different types of cytoskeleton and their functions.

### 5.4 Interaction between cytoskeleton and ECM

The interaction between the cytoskeleton and the extracellular matrix (ECM) is vital in the development of fibrosis. Fibroblast-ECM interactions, mediated by integrin receptors, convert mechanical signals from the ECM into biochemical signals that influence cell behavior. Myosin II, a motor protein, generates contractile force affecting fibroblast morphology, movement, and differentiation. In normal lung tissue, myosin II facilitates fibroblast movement and polarization. However, in fibrotic lung tissue, increased matrix stiffness activates myosin II, leading to decreased fibroblast motility and weakened polarization, while promoting differentiation into myofibroblasts. Inhibiting myosin II activity with small molecule inhibitors or siRNA can restore motility and polarization in fibrotic tissues. These findings highlight the role of matrix stiffness and myosin II in fibroblast behavior and suggest that targeting myosin II may offer new therapeutic strategies for treating fibrosis (Southern et al., 2016). During the process of skin fibrosis, intermediate filaments (IFs) play a crucial role by providing structural support and participating in cell signaling. Their stiffness is closely related to changes in the extracellular matrix, a hallmark of skin fibrosis, which involves the activation and proliferation of fibroblasts, leading to excessive deposition of extracellular matrix components like collagen. Intermediate filaments, particularly keratins, regulate cell proliferation, differentiation, and migration, which can become abnormal in fibrosis. The connection between IFs and the nucleus enables the sensing of mechanical stimuli and alters gene expression, a process that may be disrupted in fibrosis. Additionally, changes in IFs can impact inflammatory responses by influencing signaling pathways such as Rho/ROCK, YAP/TAZ, and NF-κB. Mutations in intermediate filament proteins are also linked to certain skin diseases and fibrotic disorders, highlighting their significance in skin pathology. Thus, intermediate filaments are essential for maintaining skin structure and function, and their alterations during skin fibrosis can significantly affect disease progression (Shutova and Boehncke, 2022).

## 6 Conclusion

The cytoskeleton plays a crucial role in fibrosis, affecting key processes such as cell activation, migration, collagen synthesis, cell-matrix interactions, and signal transduction. Its dynamic reorganization facilitates the activation and migration of cells to injury sites, contributing to fibrosis. The stability and dynamics of cytoskeletal components influence fibroblast function and collagen deposition, while cytoskeleton-driven cell-matrix interactions are essential for cell migration and matrix remodeling. Additionally, the cytoskeleton regulates signaling pathways like TGF-β, Wnt, and PI3K/Akt, which are pivotal in the fibrotic process. Future therapeutic strategies should focus on targeting cytoskeletal reorganization, actin and microtubule dynamics, intermediate filament functions, cell-matrix interactions, key signaling pathways, and specific cytoskeleton-associated proteins. These approaches aim to control fibrosis progression, though their efficacy requires further validation through extensive research.
